# A Case of Leg Pain After the Initial Dose of the COVID-19 Vaccine, Followed by Deep Vein Thrombosis and Pulmonary Embolism After the Second Dose

**DOI:** 10.7759/cureus.46756

**Published:** 2023-10-09

**Authors:** Michiru Nagao, Ryo Yonezawa, Daisuke Wada, Hiroshi Suzuki, Tomiko Sunaga

**Affiliations:** 1 Department of Hospital Pharmaceutics, School of Pharmacy, Showa University, Tokyo, JPN; 2 Department of Pharmacy, Showa University Fujigaoka Hospital, Kanagawa, JPN; 3 Division of Cardiology, Department of Internal Medicine, Showa University Fujigaoka Hospital, Kanagawa, JPN; 4 Department of Pharmacy, Showa University Dental Hospital, Tokyo, JPN

**Keywords:** internal medicine, naranjo scores, oral pills, d-dimer, apixaban, cardiology, infectious disease, pulmonary embolism, deep vein thrombosis, covid-19 vaccine

## Abstract

COVID-19 has been spreading worldwide. Therefore, the COVID-19 vaccine is recommended for prevention. However, adverse events after COVID-19 vaccination remain an issue, and we should monitor patients for adverse events and determine their association with COVID-19 vaccination. Here, we report a case involving a 48-year-old Japanese woman who experienced dull left leg pain that resolved spontaneously after the first vaccine dose, followed by deep vein thrombosis (DVT) and pulmonary embolism (PE) after the second dose. The findings from this case suggest that the COVID-19 vaccine could cause severe adverse events, such as DVT. Therefore, patients should understand their subjective symptoms and report any side effects experienced after the first dose before they take the second dose. Furthermore, medical providers should enquire about all possible symptoms experienced after the initial dose before they administer the second dose.

## Introduction

The COVID-19 pandemic, also known as the coronavirus pandemic, has had a severe impact worldwide. According to WHO, 400 million individuals were infected with COVID-19 until 2023 with approximately 5.7 million deaths per the latest information. Given the need for effective and safe vaccines to control the pandemic [1], the COVID-19 vaccine was developed and is recommended as a proven preventive measure. Approximately 10 billion vaccinations were administered until 2023 [2].

On the other hand, there are concerns about adverse events after COVID-19 vaccination. Kadali et al. investigated the frequency of adverse events and reported that common symptoms included soreness, fatigue, myalgia, and headache; the design and methods used in their study provided direction to assess the safety and detailed side-effect profile of mRNA-based COVID-19 vaccines [3]. The duration and severity of adverse events are not influenced by age or sex. Unusual adverse events should be carefully monitored to determine their association with the vaccine. Here, we report a case involving a Japanese woman who experienced left leg pain after the initial dose of the COVID-19 vaccine; although this resolved spontaneously, she developed deep vein thrombosis (DVT) and pulmonary embolism (PE) after the second dose.

## Case presentation

**Figure 1:**
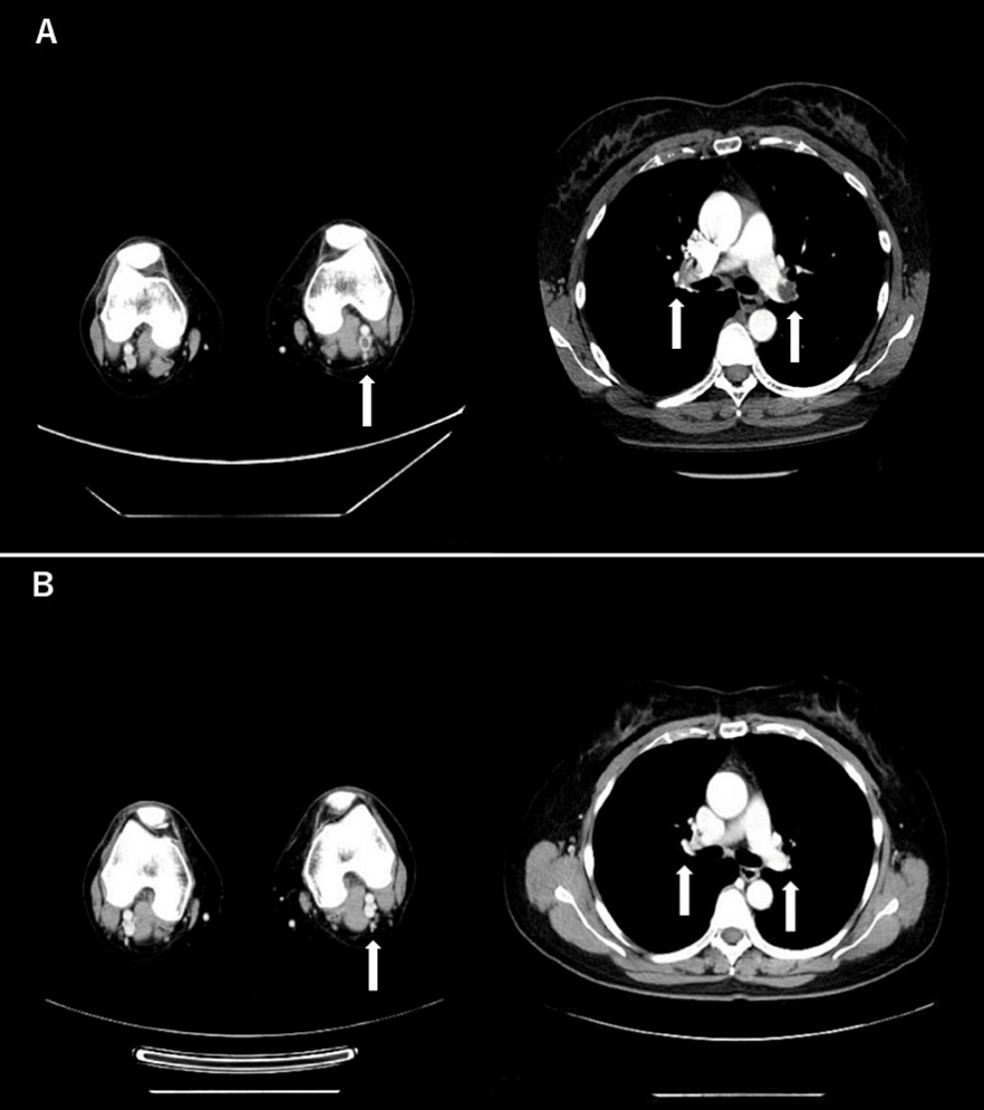
Computed tomography images taken on days 48 and 152 (day one was the day of the first COVID-19 vaccination) for a woman with deep vein thrombosis and pulmonary embolism after the second inoculation A. Contrast-enhanced CT shows thrombi in the bilateral pulmonary artery trunk and left popliteal vein on day 48. B. Contrast-enhanced CT on day 152 confirms the disappearance of the thrombi.

A 48-year-old Japanese woman developed left leg pain three days after she received the first dose of the COVID-19 vaccine (Comirnaty®, Pfizer-BioNTech, Mainz, Germany). The patient’s height, weight, and body mass index were 163 cm, 69.3 kg, and 26.1 kg/m^2^, respectively, and she had been taking a combination of norethisterone 1 mg and ethinyl estradiol 0.035 mg for dysmenorrhea for 10 years. The left leg pain spontaneously resolved in a few days, so the patient did not seek medical care. Subsequently, she received the second time dose of the vaccine (Comirnaty®, Pfizer-BioNTech, Mainz, Germany) on day 22 (day one was the day of the first dose). On day 44, she developed left leg pain with shortness of breath when climbing stairs. On day 45, the patient visited a nearby clinic and had done a blood test. A few days later, the results of the blood test arrived at the clinic, and her D-dimer level was approximately 16 μg/mL (reference value: <1 µg/mL), which was considerably high. This led to a suspicion of DVT and PE, and she was referred to our hospital on day 48. Contrast-enhanced computed tomography (CT) performed on day 48 revealed thrombi in the bilateral pulmonary artery trunks and the left popliteal vein (Figure 1A), and heparin infusion was performed as emergency treatment.

**Figure 2:**
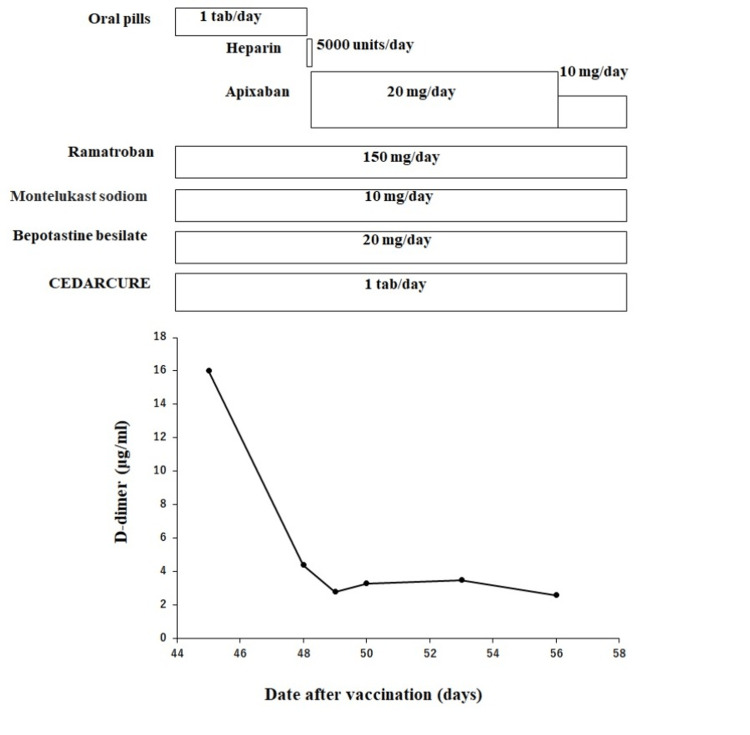
Clinical course of the case Oral pills: norethisterone 1 mg and ethinyl estradiol 0.035 mg; CEDARCURE: CEDARCURE(R)SLIT-tablets. The patient was vaccinated with a second dose of COVID-19 vaccine on day 22 and was admitted to our hospital on suspicion of DVT and PE due to a high D-dimer level. During the hospitalization period, the patient's D-dimer improved.

A blood test performed on the same day showed a high D-dimer level of 4.4 µg/mL and a C-reactive protein level of 5.5 mg/dL (reference value: <0.3 mg/dL). There was no previous record of C-reactive protein levels for comparison; however, her other clinical records showed normal values for activated partial thromboplastin time (30.1 s) and troponin I (20.3 pg/mL). Her percutaneous oxygen saturation was 98% on room air, although her heart rate was 112 beats/minute, and she still experienced shortness of breath. Her blood pressure was 151/96 mmHg, her respiratory rate was 17 breaths/minute, and her body temperature was 36.6 ℃. Left calf swelling was also observed. On the basis of her CT images and leg swelling and pain, she was diagnosed with DVT and PE and hospitalized. On admission, norethisterone 1 mg and ethinyl estradiol 0.035 mg were discontinued, and intravenous (IV) heparin 5,000 units/day was started, so we could switch to a loading dose of apixaban immediately after IV heparin administration. The reason for the change in medication was that apixaban has a shorter loading period than those of other direct oral anticoagulants. The patient received a loading dose of apixaban 20 mg/day for one week, and this was tapered to 10 mg/day for approximately six months after that. Although she experienced a slight increase in menstrual bleeding, it did not warrant cessation of the anticoagulation therapy. The patient showed subjective and biochemical improvements and was discharged home on day 57 because, given the severity of her condition, thrombi dissolution and the effects of apixaban required monitoring. Contrast-enhanced CT performed on day 152 confirmed the disappearance of the thrombi and resolution of pulmonary embolism (Figure 1B). Figure 2 shows the clinical course of this case. She was followed up in the outpatient clinic, and apixaban was discontinued approximately six months after discharge.

## Discussion

Numerous cases have been published on COVID-19-induced thrombosis, as well as thrombotic events induced by the COVID-19 vaccine [4-7]. To our knowledge, this is the report of leg pain after the first dose of the vaccine that was followed by PE and DVT after the second time dose. This highlights the importance of paying attention to adverse events after past inoculations and before subsequent inoculations.

In the present case, we distinguished between congenital and acquired risk factors and based our diagnosis on DVT guidelines [8]. Congenital factors include hyperhomocysteinemia, antithrombin deficiency, protein C deficiency, and protein S deficiency [8]. A blood test on day 48 showed normal values ​​for homocysteine ​​(11.7 nmol/mL), ATⅢ activity (93%), protein C activity (84%), and protein S activity (82%), with no correspondence with congenital factors. However, the patient had no history of long-term bed rest, blood flow stagnation such as that occurring in pregnancy, and smoking as acquired factors. Moreover, there was no increase in the blood coagulation ability, such as that observed during dehydration. The patient also showed a Well’s score of 2 and satisfied two PE rule-out criteria [8,9]. Therefore, DVT was attributed to acquired factors in this case.

Several cases of thromboembolism after COVID-19 vaccination have been reported [4-7]. Graca et al. reported that a 62-year-old woman developed symptoms such as abdominal pain, vomiting, and anemia one day after the initial dose and was found to have extensive thrombosis in the abdominal veins and arteries [4]. The patient was closely observed from the early stages of the adverse event, unlike our patient. Generally, risk factors for angiopathy include obesity, hypertension, hypertriglyceridemia, and visceral fat syndrome [5]. In our case, there were concerns about risk factors other than COVID-19 vaccination for the occurrence of adverse events; she could have been at risk of thrombosis because of long-term intake of norethisterone 1 mg and ethinyl estradiol 0.035 mg. Oral pills raise the risk of thrombosis in general. Al-Maqbali et al. reported that a 59-year-old woman who took a combination oral contraceptive comprising ethinylestradiol 30 mg+levonorgestrel 150 mg and 2,000 mg of metformin as regular medications with no interruption of therapy for 20 years experienced DVT and PE seven days after COVID-19 vaccination [6]. However, the onset of thrombosis is strongly correlated with a higher dosage and a duration of six months to one year for estrogen (50 mg) therapy. In the present case, we believe the development of thrombosis was not associated with oral pills because the patient had been taking them for 10 years at low doses. Moreover, we calculated Naranjo scores, which were 10 for COVID-19 vaccination; 7 for oral pills; and 5 for ramatroban, montelukast sodium, bepotastine besilate, and CEDARCURE(R)SLIT-tablets. Therefore, we concluded that COVID-19 vaccination might have caused DVT and PE [10].

## Conclusions

The findings from this case suggest that COVID-19 vaccination could cause serious adverse events such as DVT. Therefore, patients should understand their subjective symptoms and report any side effects experienced after the first dose before they take the second dose. Furthermore, medical providers should appropriately counsel patients and enquire about all possible symptoms experienced after the initial dose before they administer the second dose.

## References

[1] Li M, Wang H, Tian L (2022). COVID-19 vaccine development: milestones, lessons and prospects. Signal Transduct Target Ther.

[2] (2023). WHO coronavirus (COVID-19) dashboard. https://covid19.who.int.

[3] Kadali RA, Janagama R, Peruru S, Malayala SV (2021). Side effects of BNT162b2 mRNA COVID-19 vaccine: a randomized, cross-sectional study with detailed self-reported symptoms from healthcare workers. Int J Infect Dis.

[4] Graça LL, Amaral MJ, Serôdio M, Costa B (2021). Extensive thrombosis after COVID-19 vaccine: cause or coincidence?. BMJ Case Rep.

[5] Woo EJ, Mba-Jonas A, Thomas A (2022). Thromboembolic events after Ad.26.COV2.S COVID-19 vaccine: reports to the vaccine adverse event reporting system. Pharmacoepidemiol Drug Saf.

[6] Al-Maqbali JS, Al Rasbi S, Kashoub MS, Al Hinaai AM, Farhan H, Al Rawahi B, Al Alawi AM (2021). A 59-year-old woman with extensive deep vein thrombosis and pulmonary thromboembolism 7 days following a first dose of the Pfizer-BioNTech BNT162b2 mRNA COVID-19 vaccine. Am J Case Rep.

[7] Greinacher A, Thiele T, Warkentin TE, Weisser K, Kyrle PA, Eichinger S (2021). Thrombotic thrombocytopenia after ChAdOx1 nCov-19 vaccination. N Engl J Med.

[8] (2023). Diagnosis, treatment and prevention of pulmonary thromboembolism and deep vein thrombosis. https://js-phlebology.jp/wp/wp-content/uploads/2019/03/JCS2017_ito_h.pdf.

[9] Kline JA, Mitchell AM, Kabrhel C, Richman PB, Courtney DM (2004). Clinical criteria to prevent unnecessary diagnostic testing in emergency department patients with suspected pulmonary embolism. J Thromb Haemost.

[10] Naranjo CA, Busto U, Sellers EM (1981). A method for estimating the probability of adverse drug reactions. Clin Pharmacol Ther.

